# Volatilome of Chill-Stored European Seabass (*Dicentrarchus labrax*) Fillets and Atlantic Salmon (*Salmo salar*) Slices under Modified Atmosphere Packaging

**DOI:** 10.3390/molecules25081981

**Published:** 2020-04-23

**Authors:** Athanasios Kritikos, Ioanna Aska, Sotirios Ekonomou, Athanasios Mallouchos, Foteini F. Parlapani, Serkos A. Haroutounian, Ioannis S. Boziaris

**Affiliations:** 1 Laboratory of Marketing and Technology of Aquatic Products and Foods, Department of Ichthyology and Aquatic Environment, School of Agricultural Sciences, University of Thessaly, Fitoko Street, 38446 Volos, Greece; atkritik@uth.gr (A.K.); ioaska@uth.gr (I.A.); soikon@uth.gr (S.E.); fwparlap@uth.gr (F.F.P.); boziaris@uth.gr (I.S.B.); 2 Laboratory of Food Chemistry and Analysis, Department of Food Science and Human Nutrition, Agricultural University of Athens, Iera Odos 75, 118 55 Athens, Greece; 3 Department of Animal Science and Aquaculture, Agricultural University of Athens, Iera Odos 75, 118 55 Athens, Greece; sehar@aua.gr

**Keywords:** fish, seafood, modified atmosphere packaging, spoilage, shelf life, volatiles, solid phase microextraction, gas chromatography-mass spectrometry

## Abstract

Fish spoilage occurs due to production of metabolites during storage, from bacterial action and chemical reactions, which leads to sensory rejection. Investigating the volatilome profile can reveal the potential spoilage markers. The evolution of volatile organic molecules during storage of European seabass (*Dicentrarchus labrax*) fillets and Atlantic salmon (*Salmo salar*) slices under modified atmosphere packaging at 2 °C was recorded by solid-phase microextraction combined with gas chromatography-mass spectrometry. Total volatile basic nitrogen (TVB-N), microbiological, and sensory changes were also monitored. The shelf life of seabass fillets and salmon slices was 10.5 days. *Pseudomonas* and H_2_S-producing bacteria were the dominant microorganisms in both fish. TVB-N increased from the middle of storage, but never reached concentrations higher than the regulatory limit of 30–35 mg N/100 g. The volatilome consisted of a number of aldehydes, ketones, alcohols and esters, common to both fish species. However, different evolution patterns were observed, indicating the effect of fish substrate on microbial growth and eventually the generation of volatiles. The compounds 3-hydroxy-2-butanone, 2,3-butanediol, 2,3-butanedione and acetic acid could be proposed as potential spoilage markers. The identification and quantification of the volatilities of specific fish species via the development of a database with the fingerprint of fish species stored under certain storage conditions can help towards rapid spoilage assessment.

## 1. Introduction

Fish spoilage is a well-documented process resulting from chemical reactions, autolytic degradation by fish enzymes and microbial metabolic activity. However, in the chilled seafood supply chain, fresh fish quality is diminished mainly by microbial mediated changes [1,2]. Specific spoilage organisms (SSOs) is a small part of the initial total microbiota which dominate against others under particular storage conditions and produce metabolites responsible for the development of off-flavors and off-odors in fish products, thus resulting in their sensory rejection [2,3,4].

Modified atmosphere packaging (MAP) combined with low storage temperatures is an effective preservation technique to extend the shelf life of fishery products. Gas composition, temperature and fish species are some of the most important factors that influence dramatically the composition of spoilage microorganisms and eventually the produced metabolites [5]. Among the numerous metabolites produced during fish storage, volatile organic compounds (VOCs) have been the focus of several studies lately, for various reasons. Traditional spoilage indicators such as biogenic amines, total volatile basic nitrogen (TVB-N) and ATP degradation products exhibit weaknesses. Biogenic amines are not produced in considerable amounts in non-scombroid fish, while TVB-N increases in fish only at the late stages of storage, and cannot be used as spoilage/freshness markers [6]. ATP degradation products, which is a result of autolytic changes, affect sensory attributes only at the beginning of shelf life and not throughout storage period [7] and definitely does not determine fresh fish shelf life, which is a result of the accumulation of microbial metabolites [8,9]. A suitable spoilage marker should be a metabolite produced by the main spoilage microorganisms, exhibit a consistent profile, preferably increase during storage, and show satisfactory correlation with microbial growth, sensory score and remaining shelf life. Thus, volatilome and especially microbial metabolites seem to be more promising for monitoring spoilage from the beginning until the end of shelf life [10].

The method of choice for the analysis of VOCs in such studies is solid phase microextraction-gas chromatography/mass spectrometry (SPME–GC/MS) due to its simplicity and sensitivity [6,7,8,9,10]. Typical compounds associated with fish spoilage include aldehydes, ketones, alcohols, acids, amines and sulphides. Leduc et al. [11] proposed thiophene, 1-nonen-3-ol, hexanal, 1-octen-3-one and dimethyl trisulfide as markers of seabass quality. Several alcohols (cyclopentanol, *Z*-2-penten-1-ol, 1-penten-3-ol, 1-octen-3-ol) and aldehydes (hexanal, octanal, *E*-2-pentenal, *E*-2-hexenal) were identified as potential markers for salmon freshness whereas other compounds (acetoin, 3-methylbutanoic acid, acetic acid) were identified as potential markers for salmon spoilage [12]. 3-Methyl-1-butanol has been suggested as spoilage marker for ice-stored sea bream [13] and yellowfin tuna [14]. Among aldehydes, 3-methylbutanal and 2-methylbutanal have been reported as products of *Carnobacterium* species in seafood and have been found in various chilled stored fish such as sea bream [10,13], cod, whiting and mackerel [15], where *Pseudomonas* spp. and H_2_S producing bacteria predominate.

Atlantic salmon and European seabass are the two most important aquaculture fish species of the European Union and their fillets or slices are value-added products with high quality and consumer preference. Modified atmosphere packaging is a very effective way to retain fish quality for longer time than conventional refrigeration under aerobic storage. Thus, the main objectives of the present study were to (i) monitor microbiological changes and determine shelf life of seabass fillets and salmon slices stored at 2 °C under MAP with a commercial gaseous mixture used by Hellenic Aquaculture Industry, and (ii) investigate the VOCs profile related to the remaining shelf life using SPME–GC/MS, in order to identify any potential markers of spoilage/freshness.

## 2. Results and Discussion

### 2.1. Sensory Acceptance Evaluation and Shelf-Life Determination

**Figure 1:**
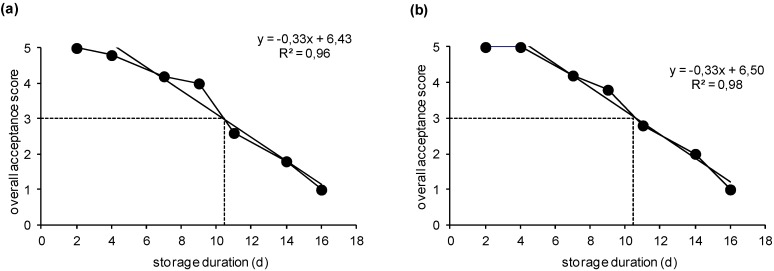
Overall acceptance scores of cooked (**a**) sea bass fillets and (**b**) salmon slices stored under modified atmosphere packaging (MAP) at 2 °C. Each data point is the mean score of 5 panelists. The cross-section of the dashed lines represents the point of minimum acceptability.

The overall sensory acceptance of cooked fish samples was evaluated, and the results are presented in Figure 1. Initially, the fish acceptance was excellent and remained at those levels for the first 4 days of storage at 2 °C under MAP (*p* > 0.05). Afterwards, the overall acceptance score diminished linearly as expressed by the regression equations. A score of 3 for overall impression was judged as the lower limit of acceptability. The time after that coincided with slight off flavor and off taste development. The shelf life of both fish products studied was estimated to be no longer than 11 days, where at least one of the panelists scored the product below 3. The shelf life of fisheries and aquaculture products depends on various factors such as the applied storage (temperature, atmosphere and packaging, e.g., gas concentration, film permeability, and headspace), transportation (storage requirements) and distribution (storage facilities, temperature) conditions and the composition and population level of the initial total microbiota (including indigenous and exogenous microbiota).

Thus, shelf life varies between fish species or even among the products (whole, gutted, fillets) [16,17]. In a recent work by our team [18], it was found that whole gutted seabass had a shelf life of 13 days, under the same conditions of temperature and atmosphere as herein. According to Kostaki et al. [19], the shelf life of sea bass fillets was 12 days when stored at 4 °C under identical gaseous composition to ours. In another study, Poli et al. [20] reported that the shelf life of seabass fillets stored at 2 °C under MAP was 8 days. However, they used a different gas atmosphere. Similarly, variable results have been reported for salmon fillets stored under MAP [21,22,23].

### 2.2. Microbiological Changes

**Figure 2:**
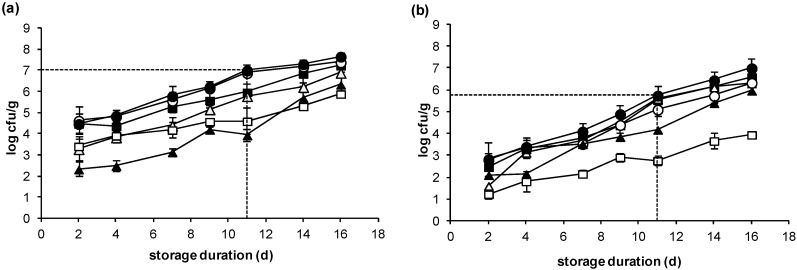
Microbiological changes during storage of (**a**) sea bass fillets and (**b**) salmon slices at 2 °C under MAP. Total viable count (●), *Enterobacteriaceae* (□), *Brochothrix thermosphacta* (▲), *Pseudomonas* spp. (○), Lactic acid bacteria (Δ) and H_2_S producing bacteria (■). Each data point and the error bars show the mean ± standard deviation of 4 replicates. The cross-section of dashed lines indicates the point of sensory rejection (11 days).

After 2 days of storage, the total microbial population of seabass fillets expressed by total viable count (TVC) was at the level of 4.5 log cfu/g. At the time of sensory rejection, the total microbial population reached 6.8 log cfu/g (Figure 2a). Initial (day 2) microbial populations of spoilage bacteria were at the level of 3.3, 4.6, 4.5, 3.4 and 2.3 log cfu/g, for lactic acid bacteria (LAB), *Pseudomonas* spp., H_2_S producing bacteria (presumable *Shewanella* spp.), *Enterobacteriaceae* and *B. thermosphacta*, respectively. The dominant microorganisms were *Pseudomonas* spp., reaching at the end of shelf life populations densities as high as 6.7 log cfu/g (*p* < 0.05), followed by H_2_S producing bacteria and LAB with 5.9 log cfu/g, approximately. *B. thermosphacta* and *Enterobacteriaceae* populations were not higher than 4.6 log cfu/g. These results are in agreement with our previous work [18] conducted under the same conditions with whole gutted seabass. In all cases, our previous and present study, it was noticed that *Pseudomonas* was the most dominant microorganism, possibly due to the use of a high O_2_ concentration level (10%). On the other hand, *B. thermosphacta* and LAB populations usually predominate under reduced O_2_ and elevated CO_2_ of MAP by outcompeting the strictly aerobic *Pseudomonas* spp. [24]. However, this was not observed in the present work, probably due to different gas composition. Indeed, the study of Parlapani et al. [25], using molecular techniques found that *Pseudomonas* spp. was still a great part of spoilage microbiota together with *Carnobacterium* spp. and other LAB in gilt-head seabream fillets stored in the same packaging conditions.

Regarding salmon slices, the total microbial load did not exceed 3 log cfu/g after 2 days of storage, whereas at the end of shelf life reached the level of 5.6 log cfu/g (Figure 2b). In contrast to seabass fillets, the dominant microorganisms were H_2_S producing bacteria and LAB that reached a value of 5.3 log cfu/g, followed by *Pseudomonas* spp. with 4.9 log cfu/g (*p* > 0.05). *B. thermosphacta* and *Enterobacteriaceae* counts were significantly lower (*p* < 0.05) at the sensory rejection point, reaching the values of 4.1 and 2.8 log cfu/g, respectively. Similar observations have been reported by Powell and Tamplin [26], who highlighted the importance of LAB in fresh Atlantic salmon stored under MAP. Using culture-independent methods, they found that the microbial communities were dominated by *Shewanella* spp. and *Carnobacterium* spp., after 15 days of storage. The low spoilage level of 5.8 log cfu/g might be due to the fact that other mechanisms, such as lipid oxidation of salmon, which is quite fatty fish compared to seabass, occurred taken into account the 10% of oxygen used in this package.

Between the two fish species, LAB and *B. thermosphacta* counts were the same (*p* > 0.05) within each sampling day. *Enterobacteriaceae* and *Pseudomonas* spp. counts were always higher in seabass fillets. H_2_S bacteria counts were significantly higher in seabass during the 7 days of storage, but afterwards, their levels did not differ greatly (*p* > 0.05) between salmon and sea bass.

### 2.3. TVB-N Determination

**Figure 3:**
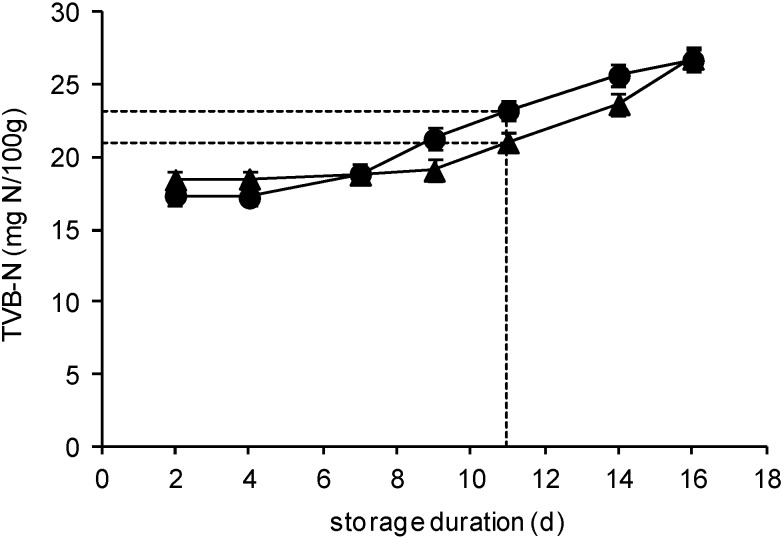
TVB-N changes of sea bass fillets (▲) and salmon slices (●) stored under MAP at 2 °C. Each data point and the error bars show the mean ± standard deviation (mg N/100 g) of 4 replicates. The cross-section of the dashed lines represents the point of sensory rejection (11 days).

Figure 3 presents the changes of TVB-N during the storage of seabass fillets and salmon slices under MAP at 2 °C. During the first 9 days of storage, the TVB-N values of seabass fillets were similar (*p* > 0.05). At the sensory rejection time point, the concentration of TVB-N (20.5 mg N/100 g) increased significantly (*p* < 0.05), reaching the value of 26.8 mg N/100 g at the end of storage period.

On the contrary, the TVB-N amount of salmon slices remained practically constant (*p* > 0.05) for the first 4 days of storage and then it increased significantly until the end of storage. At the sensory rejection time point, it reached the value of 22.7 mg N/100 g. The TVB-N values observed were similar to those reported in the literature for either seabass [27,28] or salmon [29].

At the end of shelf life, TVB-N values never reached the legislated regulatory limit, which is at 30–35 mg N/100 g, [30]. It has already been shown that this parameter—often used as a spoilage quality indicator for seafood kept on ice—displays lower values for fish stored in a CO_2_ atmosphere and considered spoiled by sensory analysis [17]. Therefore, TVB-N should be considered as a poor indicator of fish freshness, as also proposed by others [27,28].

### 2.4. Production of Volatile Compounds During Fish Storage

The analysis by SPME-GC/MS of the salmon slices and seabass fillets at different storage stages under MAP at 2 °C identified 54 volatile compounds (excluding hydrocarbons, terpenoids and miscellaneous compounds) that were classified by their characteristic functional group (Table 1).

The majority of them were mainly aldehydes (22), followed by alcohols (13), ketones (12), esters (6), and one acid (acetic acid), which were all found in the two species studied except 2,3-butanediol, 3-hydroxy-2-butanone (acetoin) and ethyl lactate. The first two compounds were detected only in seabass fillets whereas ethyl lactate was found only in salmon. By comparing the relative concentrations of the compounds at different stages of storage (day 2, 7, 11, 14), it was possible to identify compounds whose levels increased, decreased or fluctuated during spoilage. Interestingly, the behavior during storage was similar within certain classes of compounds but different between fish species, as it will be further discussed.

Among the numerous aldehydes detected in seabass fillets, the amounts of the higher members of saturated aldehydes (nonanal, decanal, undecanal), unsaturated aldehydes with 8, 10, 11 carbon atoms (2-octenal, 2-decenal, 2-undecenal), aromatic aldehydes (benzaldehyde, phenylacetaldehyde) as well as 2,4-decadienal increased by 66% from the middle stages of storage (day 7) until the last sampling point (day 14). Due to their similar evolution profile, they are referred hereafter as group Ald-1 (Table 1). A different trend was observed for the rest aldehydes, such as the saturated homologues with 2–8 carbon atoms and the unsaturated ones with 5–7 carbon atoms (group Ald-2). Their levels remained approximately constant during the first seven days of storage, then reached a maximum at the rejection point (66% increase) and subsequently, they declined by a factor of 50%.

These two patterns of volatiles’ evolution during storage of seabass fillets under MAP at 2 °C were also observed for other classes of chemical compounds, such as ketones (group Ket-1 and Ket-2), alcohols (Alc-1 and Alc-2) and esters (Est-1 and Est-2). The Ket-1 group comprised mainly of compounds with the carbonyl group at the 2-position (2-butanone, 2-heptanone, 6-methyl-5-hepten-2-one, 2-nonanone), except from 2-pentanone, which revealed a profile similar to that of C5 and C8-diones (2,3-pentanedione, 2,3-octanedione) and 3,5-octadien-3-one isomers (group Ket-2). The majority of alcohols (group Alc-2) exhibited a pattern similar to Ald-2 group. Among them, 1-penten-3-ol, 1-octen-3-ol, hexanol and octa-1,5-dien-3-ol were found in greater amounts. On the other hand, 2-ethyl-1-hexanol and dodecanol were the only members of group Alc-1. Although esters were detected at very low levels, it is noteworthy that all ethyl esters of short chain fatty acids (C4-C10) exhibited the same profile (group Est-2). An exemption was ethyl acetate (Est-1), whose relative concentration increased by 90% from day 7 to day 14.

**Figure 4:**
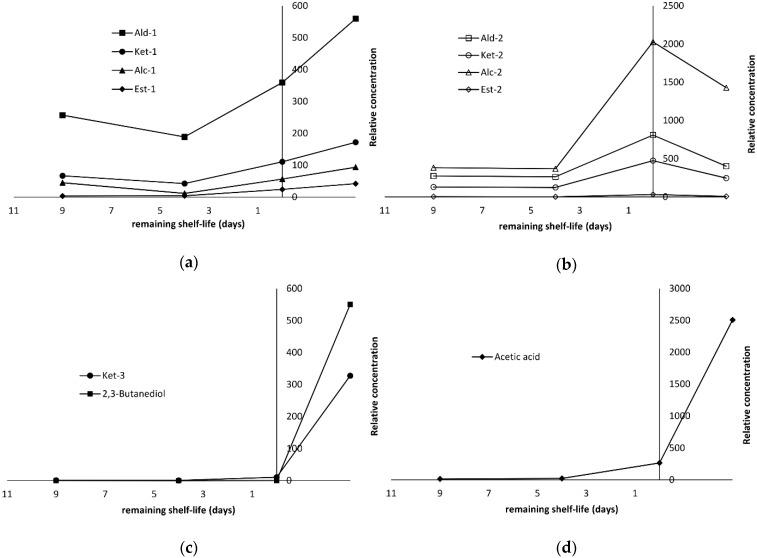
Changes of volatiles in relation to the remaining shelf life during storage of sea bass fillets under MAP at 2 °C: (**a**) Group Ald-1, Ket-1, Alc-1 and Est-1; (**b**) Group Ald-2, Ket-2, Alc-2 and Est-2; (**c**) Group Ket-3 and 2,3-butanediol; (**d**) Acetic acid. The compounds included in each group are described in Table 1.

Since the focus of the present study was to find volatiles suitable as spoilage markers, the relationships of the aforementioned evolution profiles with the remaining shelf life were depicted schematically (Figure 4a,b).

As it can be seen, when the fish was considered fresh (remaining shelf life over 7 days), the amounts of aldehydes, ketones, alcohols and esters groups remained relatively low. However, at the rejection point (0 days of remaining shelf life), their respective amounts increased substantially. The most remarkable evolution patterns were observed for 2,3-butanedione, 3-hydroxy-2-butanone (group Ket-3) and acetic acid (Table 1, Figure 4c,d). Their amounts were negligible initially, when the fish remaining shelf life was over 5 days. Their relative concentration started to increased and subsequently reached a maximum at the end of shelf life (0 remaining days), corresponding to an almost 100% increase. 2,3-Butanediol was the only compound that was not detected until day 11, which is the end of shelf life, but reached a high concentration at the end of storage. The formation of the aforementioned compounds has been associated with microbial activity occurring during storage. 2,3-Butanedione is reduced to acetoin, which is in turn reduced to 2,3-butanediol through enzymatic mediated reaction [31]. Acetoin formation in seafood has been associated mainly with LAB [32] and in some cases to *Photobacterium phosphoreum* [33] and *Shewanella baltica* [34], whereas in meat has been reported for *Pseudomonas* spp. [35]. The production of acetic acid has been associated with the metabolic activity of *B. thermosphacta*, some heterofermentative LAB and *Shewanella* spp. [34,36,37,38]. In a previous study [39], these compounds were attributed exclusively to microbial activity, as they were detected only in inoculated sterile fish juice. According to our results, these four compounds (diacetyl, acetoin, 2,3-butanediol, acetic acid) may be suggested as spoilage markers of seabass stored under MAP.

**Figure 5:**
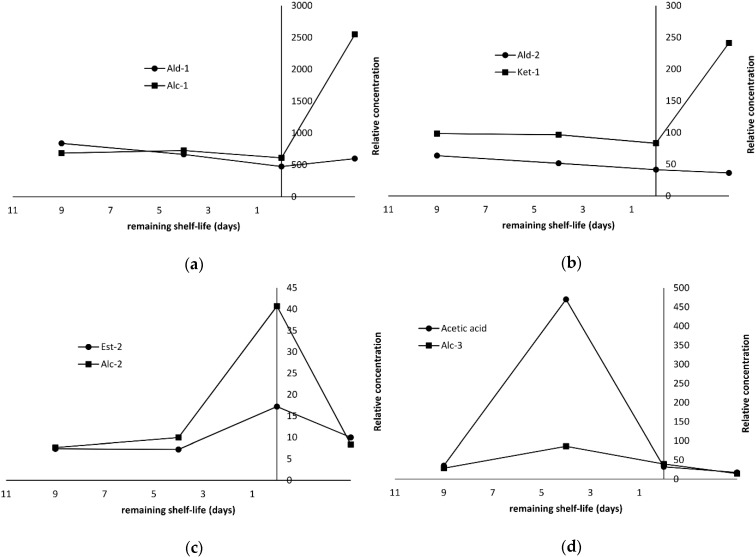
Changes of volatiles in relation to remaining shelf life during storage of salmon slices under MAP at 2 °C. (**a**) Group Ald-1 and Alc-1; (**b**) Group Ald-2 and Ket-1; (**c**) Group Est-2 and Alc-2; (**d**) Group Alc-3 and Acetic acid. The compounds included in each group are described in Table 1.

As with seabass, aldehydes dominated the volatile fraction of salmon slices stored under MAP at 2 °C. The most abundant compounds were hexanal and nonanal followed by acetaldehyde (Table 1). Among aldehydes, two groups having different evolution patterns can be distinguished. The first group (Ald-1) includes the saturated homologues with 5–10 carbon atoms (pentanal to decanal) and three unsaturated members, namely 2-pentenal, 2-hexenal and 4-heptenal. Their amounts decreased from day 2 to day 11 and afterwards they increased or remained constant. This was more pronounced with the unsaturated members, whose relative amounts were found 50–60% lower at day 11. On the contrary, the Ald-2 group, which comprised of 2-alkenals with 7–11 carbon atoms (2-heptenal, 2-octenal, 2-decenal, 2-undecenal) as well as 2,4-decadienal, followed an almost linear declining trend throughout storage. This is depicted clearly in Figure 5a,b relatively to the remaining shelf life. This linear trend can be exploited as a potential freshness index for salmon stored under MAP.

Among the detected ketones, the 2-alkanones (2-butanone, 2-pentanone, 2-heptanone, 2-nonanone) as well as 2,3-octanedione and the two 3,5-octadien-2-one isomers presented a distinct evolution pattern (Ket-1 group, Table 1). Their levels remained constant or decreased slightly during the first 11 days of storage. After the sensory rejection point (0 days of remaining shelf life), the relative amount of Ket-1 group increased by 150%, (Figure 5b). This characteristic profile was also observed for Alc-1 group, which comprised mainly of 1-octen-3-ol, (5*Z*)-octa-1,5-dien-3-ol and 1-penten-3-ol (Figure 5a). The latter one was the main alcohol produced during spoilage besides ethanol, whose amount fluctuated. Hexanol, 3-methyl-1-butanol (Alc-2 group) and ethyl esters of C6-C10 fatty acids (Est-1 group) followed a pattern (Figure 5c) similar to that in seabass (Figure 4b). The levels of acetic acid and group Alc-2 (propanol and dodecanol) presented a maximum before the rejection point (0 days of remaining shelf life), and then they declined rapidly (Figure 5d). However, the observed profile of acetic acid in salmon was different from the respective one in seabass. Furthermore, it is in contrast to other researchers who suggested acetic acid as a spoilage marker in fresh king salmon [12] and salmon fillets [29].

A notable difference between the examined fish species was the absence of acetoin and 2,3-butanediol in the salmon samples. This is contradictory to the results reported by Wierda et al. [12] albeit the storage conditions were different from ours. Furthermore, a small amount of ethyl lactate was detected only in salmon, but its levels fluctuated during storage (Table 1).

Most of the VOCs detected in this study have also been reported for other fish and seafood by other researchers as well [11,13,14,15,32,40,41]. It is generally known that most of the saturated or unsaturated aldehydes, alcohols and carbonyls in fish flesh come from the autoxidation of the polyunsaturated fatty acids resulting in the formation of hydroperoxides. Although, autoxidation of fatty acids can be initiated by a catalyst such as light or oxygen or by enzymes coming from the fish flesh, it can be also initiated by the enzymes of the microorganisms present in fish [42]. Thus, we can infer that the different evolution patterns observed between the two fish species could be attributed both to their characteristic microbiota growth as discussed earlier and the lipid composition of each fish. It is known that different fish species from different geographical areas are spoiled by different SSOs, even if the fish are stored under identical conditions (temperature and atmosphere), which means that different metabolites might be produced (9). This can explain the difference on VOCs profile between the two different fish. Various alcohols, aldehydes and esters, such as 3-methyl-1-butanol, 3- and 2-methylbutanal and ethyl esters of short chain fatty acids (C4-C10) have been suggested as potential spoilage indicators in fish and meat products [10,13,32,39,43,44,45]. However, our results from both fish species studied, indicate that they were produced (if any) at very low levels during storage. Thus, their usefulness as biomarkers may be limited under MAP in combination with low temperatures.

## 3. Materials and Methods

### 3.1. Fish Provision, Handling and Storage

Seabass fillets and salmon slices were obtained directly from a Greek fish processing plant (Selonda Aquaculture SA, Magoula, Attica, Greece). Seabass was farmed in the geographical area designated as FAO 37, 3.1 (Aegean Sea, Greece), while salmon was farmed in the European Union (Norway) and was imported to Greece. The products were packed under MAP in polysterene trays (Sirap Gema S.p.A., Verolanuova, Italy) covered with a MAP film (BDF 8050F, Cryovac-Sealed Air Ltd., Athens, Greece). Each tray contained either two seabass fillets (approximately 120 g each fillet) or one salmon slice (approximately 200 g). The concentrations of gases were CO_2_: 60%, O_2_: 10%, N_2_: 30% as recommended by the Hellenic seafood industry for this type of products. The products were delivered to the laboratory within 5 h after packaging (day 0) in insulated boxes with ice flakes. Subsequently, the products were stored in incubators (Panasonic MIR-254 cooled incubator, PHC Europe B.V., Etten-Leur, The Netherlands) operating at 2 °C. The two batches (lots) of each product were provided with 15 days difference in June 2016. The sampling started the day after receiving the products (day 1). At each sampling point, a suitable quantity of product (sample) was taken from 2 different packages for each batch (lot) of product (2 replicate samples per batch) and analyzed as described below. Thus, four replicate determinations were accomplished at each sampling point (*n* = 4 = 1 sample/package × 2 packages/batch × 2 batches), except for volatiles, where the samples were pooled as described in VOCs analyses section.

### 3.2. Sensory Acceptance Evaluation

The attributes of cooked fish (flavor and taste) were evaluated by five trained members of our Department (Dept. Ichthyology & Aquatic Environment, University of Thessaly, Greece). Approximately 20 g of fish flesh were removed, wrapped in aluminum foil and cooked in an oven preheated at 180 °C for 20 min. After that, the cooked samples were left to cool and evaluated. The panel was asked to evaluate overall impression and acceptability. Rating was assigned on a 1–5 descriptive hedonic scale (5 = like extremely, 4 = like, 3 = neutral, 2 = dislike and 1 = dislike extremely). A score of 3 was considered as the score for minimum acceptability, hence the time point corresponded to a score below 3 was set as end of shelf life. Only cooked samples were chosen to be evaluated since the aim of the sensory acceptance evaluation was to determine the shelf life of fish products and correlated with the production of various potential freshness/spoilage markers and for this reason an in-depth analysis of sensory descriptors changes of raw samples was not performed.

### 3.3. Microbiological Analysis

A sample of 25 g fish product (representing 1 replicate as described in Section 3.1) was placed into a stomacher bag containing 225 mL sterile MRD (Maximum Recovery Diluent, 8.5 g/L NaCl, 1.0 g/L bacteriological peptone) and homogenized for 1 min using a Stomacher (Bug Mixer, Interscience, London, UK). Then, 0.1 mL of 10-fold serial dilutions in MRD were used for the spread plate technique for the enumeration of the following microorganisms: (a) *Pseudomonas* spp. on cetrimide-fucidin-cephaloridine agar (CFC, LAB M, Lancashire, UK), and (b) *Brochothrix thermosphacta* on streptomycin sulphate, thallus acetate, cycloheximide (actidione) agar (STAA, Biolife Italiana srl, Milano, Italy), after incubation at 25 °C for 48 h. Additionally, 1 mL of the serial dilution were used for the pour plate with overlay technique for the enumeration of (a) the Total viable counts (TVC) on Iron Agar (IA, prepared according to Gram et al. 1987, by mixing ingredients obtained from LAB M, Lancashire, UK), and H_2_S producing bacteria (presumable *Shewanella* spp.) on IA by counting only the black colonies, after incubation at 25 °C for 72 h, (b) *Enterobacteriaceae* on Violet Red Bile Glucose agar (VRBGA, LAB M, Lancashire, UK), after incubation at 37 °C for 24 h and (c) Lactic Acid Bacteria (LAB, LAB M, Lancashire, UK) on De Man, Rogosa, Sharpe agar (MRS, LAB M, Lancashire, UK) after incubation at 25 °C for 72 h. All plates were incubated aerobically. The results were expressed as mean log cfu/g ± standard deviation of 4 replicate samples (2 replicates per batch of fish product).

### 3.4. Determination of TVB-N

A sample of 10 g fish product (representing 1 replicate as described in Section 3.1) was homogenized in trichloroacetic acid solution (TCA 60 g/L), filtered through Whatman No.1 paper in a 100 mL volumetric flask and made up to volume with TCA solution. An aliquot of 50 mL extract was analyzed for TVB-N using the steam-distillation procedure [46]. Each extract was analyzed twice, and the mean value was used for further calculations. The results were expressed as mean mg N/100 g ± standard deviation of 4 replicate samples (2 replicates per batch of fish product). All chemical reagents were supplied by Sigma-Aldrich (Steinheim, Germany).

### 3.5. VOCs Determination by Headspace SPME-GC/MS

At each sampling point, a total amount of 50 g fish product was obtained from 4 different packages (2 packages from each batch) and pooled. Then, 5 g of the pooled fish sample, 5 mL of 30% NaCl solution and 100 μL of internal standard (4-methyl-1-pentanol, in-vial concentration 1000 μg/L) were transferred into a 20 mL glass vial and homogenized with a glass rod for 1 min. The vial was hermetically closed with a Mininert valve (Sigma Aldrich, St. Louis, MO, USA) and headspace SPME-GC/MS analysis was performed according to Parlapani et al. [18]. Identification of the compounds was performed by comparing: (i) the retention indices (RI) based on an homologous series of even numbered *n*-alkanes (C8–C24, Polyscience, Niles, IL, USA) with those of authentic compounds and by comparison with literature data, and (ii) MS data with those of reference compounds and by MS data obtained from NIST 14 (NIST/EPA/NIH Mass Spectral Library with Search Program, software version 2.0d, Gaithersburg, MD, U.S.) and WILEY 7 libraries. AMDIS software (version 2.62, http://chemdata.nist.gov/mass-spc/amdis/, Gaithersburg, MD, U.S.) was used for the deconvolution of mass spectra and identification of target components. The volatile compounds were quantified by dividing the peak area of the compound of interest by the peak area of internal standard (IS) and multiplying this ratio by the concentration of the IS (expressed as μg/L). The peak areas were measured by selecting single ions (Table 1). Each pooled fish sample was extracted and analysed twice and the mean values were used. All authentic compounds used were of analytical grade and purchased from Sigma Aldrich (Steinheim, Germany).

### 3.6. Statistical Analysis

The t-test of means or Analysis of Variance followed by Tukey′s significant difference test, using STATISTICA 6.0 (Stat Soft Inc., Tulsa, OK, U.S.), were used to compare the means in viable counts, TVB-N and overall sensory acceptance score. A probability level of *p* ≤ 0.05 was considered statistically significant. Statistical analysis of VOCs was not performed, for the reason that the measurements were conducted in duplicates from a pooled sample, since the aim of the study was to monitor the profile of VOCs evolution during storage and distribution and reveal any potential spoilage marker.

## 4. Conclusions

The same storage conditions, MAP and temperature, affected the growth of microbial populations in a completely different way between seabass and salmon, thus affecting the evolution of fish volatiles. This reflects the difficulty of defining common markers of fish spoilage or freshness among fish species. It is known that different spoilage microorganisms are grown on fish from different geographical areas, even if these fish are stored under the same conditions, hence different microbial origin VOCs are produced. Therefore, in seeking potential spoilage markers using the volatilome, the research should be focused on the following directions: a) identification and quantification of species specific volatile markers under specified storage conditions or b) exploration of the overall trend of volatilome through multivariate data analysis and development of a large database with the volatile fingerprint of each fish species produced in certain storage conditions.

## Figures and Tables

**Table 1:** Relative concentrations ^a^ of volatile compounds in sea bass fillets and salmon slices during storage under MAP at 2 °C.

Compound	Sea Bass Fillets					Salmon Slices							
	D2	D7	D11	D14	Group ^b^	D2	D7	D11	D14	Group	Identification ^c^	RI ^d^	*m/z* ^e^
**Aldehydes**												
Acetaldehyde	12.0	11.5	43.0	nd ^f^	Ald-2	128	62.5	77.8	61.5		MS, RI, ref	459	29
3-Methylbutanal	nd	nd	3.1	nd	Ald-2	5.2	9.3	5.1	6.9		MS, RI, ref	647	58
2-Methylbutanal	nd	nd	2.9	nd	Ald-2	4.7	4.9	4.0	3.1		MS, RI, ref	657	57
Pentanal	10.4	9.3	33.1	12.0	Ald-2	21.9	24.4	9.0	23.1	Ald-1	MS, RI	697	58
Hexanal	132	152	357	164	Ald-2	251	157	120	159	Ald-1	MS, RI, ref	802	56
Heptanal	19.6	29.3	45.9	42.9	Ald-2	53.9	33.7	31.4	36.1	Ald-1	MS, RI	902	70
Octanal	31.6	19.5	46.3	43.2	Ald-2	28.7	20.5	18.5	23.1	Ald-1	MS, RI, ref	1003	43
Nonanal	124	115	160	214	Ald-1	216	198	145	150	Ald-1	MS, RI	1105	57
Decanal	35.6	14.0	42.7	61.1	Ald-1	31.9	26.6	24.4	29.5	Ald-1	MS, RI, ref	1206	57
Undecanal	4.5	3.7	8.6	12.1	Ald-1	5.8	7.6	4.2	5.3		MS, RI	1308	57
(*E*)-2-Pentenal	6.8	3.9	25.4	13.9	Ald-2	10.7	15.9	3.9	7.4	Ald-1	MS, RI	748	55
(*E*)-2-Hexenal	3.4	1.4	13.8	9.4	Ald-2	5.5	12.4	2.8	5.8	Ald-1	MS, RI	853	83
4-Heptenal	12.5	6.9	51.4	20.7	Ald-2	8.8	14.6	4.0	33.1	Ald-1	MS, RI	901	84
2-Heptenal	4.2	2.7	10.3	8.6	Ald-2	8.3	6.4	4.6	4.5	Ald-2	MS, RI	954	83
(*E*)-2-Octenal	8.6	5.5	12.0	20.0	Ald-1	12.5	8.6	7.6	6.0	Ald-2	MS, RI	1060	70
(*E*)-2-Decenal	10.5	7.0	12.6	28.6	Ald-1	15.8	14.0	11.8	9.9	Ald-2	MS, RI	1262	55
(*E*)-2-Undecenal	10.6	7.0	14.0	29.7	Ald-1	15.2	14.2	10.5	9.3	Ald-2	MS, RI	1365	70
2,4-Heptadienal (isomer)	17.7	11.6	81.6	33.8	Ald-2	28.5	40.6	5.4	21.5		MS	998	81
(*E,E*)-2,4-Heptadienal	23.4	15.0	94.2	53.9	Ald-2	27.9	28.8	13.4	28.0		MS, RI	1011	81
2,4-Decadienal	8.7	5.6	15.5	24.8	Ald-1	11.7	8.3	6.9	6.7	Ald-2	MS, RI	1315	81
Phenylacetaldehyde	8.9	5.4	18.1	41.0	Ald-1	13.4	26.7	13.4	17.9		MS, RI	1043	91
Benzaldehyde	46.2	25.4	75.6	127.6	Ald-1	47.0	49.4	31.5	53.5		MS, RI, ref	955	106
**Ketones**													
2,3-Butanedione	1.0	0.6	5.7	69.2	Ket-3	5.6	5.0	6.8	5.0		MS, RI, ref	594	86
2-Butanone	12.0	7.6	26.1	28.7	Ket-1	19.8	17.1	21.1	32.7	Ket-1	MS, RI	601	72
2-Pentanone	28.3	17.0	35.3	29.0	Ket-2	24.8	16.5	22.5	66.7	Ket-1	MS, RI	686	43
2,3-Pentanedione	20.5	17.1	65.6	38.0	Ket-2	63.9	78.6	34.4	50.7		MS, RI, ref	696	100
3-Hydroxy-2-butanone	nd	nd	4.8	258	Ket-3	nd	nd	nd	nd		MS, RI, ref	715	45
2-Heptanone	9.9	5.4	20.3	25.7	Ket-1	5.2	4.8	8.0	13.2	Ket-1	MS, RI	888	43
2,3-Octanedione	28.7	44.9	107	47.0	Ket-2	17.2	15.6	14.8	42.7	Ket-1	MS, RI	987	43
6-Methyl-5-hepten-2-one	9.5	7.4	12.5	15.8	Ket-1	7.8	8.7	7.4	5.4		MS, RI	989	108
(*E,E*)-3,5-Octadien-2-one	42.3	36.9	216	106	Ket-2	28.2	37.7	7.5	50.6	Ket-1	MS, RI	1072	95
2-Nonanone	10.8	4.9	18.9	30.3	Ket-1	3.3	4.9	5.1	15.4	Ket-1	MS, RI	1094	58
3,5-Octadien-2-one (isomer)	8.4	7.4	49.7	26.1	Ket-2	nd	nd	4.1	19.7	Ket-1	MS, RI	1094	95
Acetophenone	24.8	17.2	32.9	71.4	Ket-1	13.9	19.1	10.7	17.1		MS, RI, ref	1065	105
**Alcohols**													
Ethanol	478	111	1336	567	Alc-2	1294	482	1151	505		MS, RI, ref	477	45
Propanol	12.4	10.8	54.1	8.4	Alc-2	14.8	62.4	28.7	9.7	Alc-3	MS, RI, ref	555	31
3-Methyl-1-butanol	nd	nd	1.8	nd	Alc-2	nd	nd	6.3	nd	Alc-2	MS, RI, ref	725	55
Pentanol	9.5	8.5	30.1	23.4	Alc-2	11.8	11.3	13.0	11.4		MS, RI, ref	759	42
Hexanol	21.7	24.8	88.9	48.6	Alc-2	7.7	10.0	34.4	8.4	Alc-2	MS, RI, ref	870	56
Heptanol	6.3	5.8	22.0	15.3	Alc-2	7.9	4.9	6.8	6.1		MS, RI, ref	973	70
2-Ethyl-1-hexanol	40.2	7.7	49.4	81.8	Alc-1	15.7	14.2	19.4	45.4	Alc-1	MS, RI, ref	1030	57
Dodecanol	4.9	3.7	7.2	11.9	Alc-1	13.7	23.5	10.8	4.9	Alc-3	MS, RI	1476	55
1-Penten-3-ol	145	148	746	600	Alc-2	552	548	474	1468	Alc-1	MS, RI, ref	675	57
(*Z*)-2-Penten-1-ol	29.3	27.5	160	148	Alc-2	70.0	108	32.7	108		MS, RI	766	57
1-Octen-3-ol	52.1	53.3	374	196	Alc-2	23.7	37.0	33.7	212	Alc-1	MS, RI, ref	980	57
2,3-Butanediol	nd	nd	nd	550	Alc-3	nd	nd	nd	nd		MS, RI	794	45
(5*Z*)-Octa-1,5-dien-3-ol	106	88.9	551	389	Alc-2	93.1	124	83.4	821	Alc-1	MS, RI	975	57
**Esters**													
Ethyl acetate	3.4	4.1	24.3	42.1	Est-1	13.2	7.3	17.0	5.4		MS, RI, ref	613	61
Ethyl butanoate	nd	nd	4.5	nd	Est-2	17.4	5.0	15.6	6.9		MS, RI, ref	805	88
Ethyl lactate	nd	nd	nd	nd		27.0	5.6	21.2	4.7		MS, RI, ref	815	45
Ethyl hexanoate	1.8	0.8	19.4	3.0	Est-2	7.3	7.2	12.8	6.8	Est-1	MS, RI, ref	1001	88
Ethyl octanoate	1.1	nd	7.2	3.3	Est-2	nd	nd	3.1	2.4	Est-1	MS, RI, ref	1198	88
Ethyl decanoate	nd	nd	1.8	nd	Est-2	nd	nd	1.3	0.8	Est-1	MS, RI, ref	1396	88
**Acids**													
Acetic acid	13.5	22.3	266	2507		35.4	470	32.2	17.4		MS, RI, ref	624	60

^a^ Each value is the mean of duplicate measurements of pooled samples. Expressed as the ratio of each compound peak area to that of internal standard multiplied by its concentration (1000 μg/L). ^b^ Within each fish species, compounds belonging to the same group presented similar evolution pattern during storage. ^c^ Experimental retention indices on HP-5MS column. ^d^ Identification confirmed by MS, mass spectra; RI, retention indices provided with NIST14 mass spectral library; ref, identified by comparison to authentic compound. Unless confirmed by comparison to authentic standards, compounds are considered as tentatively identified. ^e^ Mass fragment used in peak area calculation. ^f^ Not detected.
